# Preparation and characterization of novel flame-retardant paint of substituted cyclodiphosph(V)azane sulfonomide and their Cu(II), Cd(II) metal complexes as new additives for exterior wood coating protection

**DOI:** 10.1038/s41598-024-64065-w

**Published:** 2024-06-24

**Authors:** Narmeen G. El khashab, Salwa A. H. Albohy, H. Abd El-Wahab, Moustafa M. G. Fouda, Carmen M. Sharaby

**Affiliations:** 1 Chemistry Department, Faculty of Science (Girls), Al-Azhar University, Nasr City, Cairo, Egypt; 2 Chemistry Department, Faculty of Science (Boys), Al-Azhar University, Nasr City, Cairo, Egypt; 3 Pre-Treatment and Finishing of Cellulose Based Textiles, Textile Research and Technology Institute (TRT), National Research Center, 33-El-Buhouth St, Dokki, Cairo, Egypt

**Keywords:** Hexachlorocyclodiphosph(v)azane, Transition metal complexes, Flame-retardant paint, Molecular docking, Chemistry, Analytical chemistry, Biochemistry

## Abstract

The development of flame-retardant materials has become an important research direction. For the past dozen years, researchers have been exploring flame retardants with high flame-retardant efficiency, low toxicity, less smoke, or other excellent performance flame retardants. Therefore, this work aimed to synthesize new cyclodiphosph(V)azane derivatives and their Cu(II) and Cd(II) metal complexes and investigated their potential applications as high flame-retardant efficiency. Various techniques were used to characterize the prepared ligand H_2_L and its metal complexes, including elemental analyses, mass spectra, conductivity measurements, electronic spectral data UV–vis, FT-IR, ^1^H,^13^C-NMR, TGA, XRD, and molecular docking experiments studies were *M. tuberculosis* receptors (PDB ID: 5UHF) and the crystal structure of human topoisomerase II alpha (PDB ID: 4FM9). Wood-based paint was physically mixed with the ligand H_2_L and its metal complexes. The obtained results of mechanical characteristics of the dried paint layers were noticed to improve, such as gloss value, which ranged from 85 to 95, hardness 1.5–2.5 kg, adhesion 4B to 5B, and impact resistance, which improved from 1.3 to 2.5 J. Moreover, the obtained results of flame-retardant properties showed a significant retardant impact compared to the blank sample, such as ignitability, which includes the heat flux which increased from 10 to 25 kW/m^2^, and ignition time, ranging from 550 to 1200 s, respectively, and limiting oxygen index (LOI) (%) which has been increased from 21 to 130 compared with the plywood sample and sample blank. The ordering activity of the observed results was noticed that coated sample based on Cd(II) metal complexes > coated sample based on Cu(II) metal complexes of Cyclophosphazene ligand > coated sample based on phosphazene ligand H_2_L > coated sample without additives > uncoated sample. This efficiency may be attributed to (1) the H_2_L is an organophosphorus compound, which contains P, N, Cl, and aromatic six- and five-member ring, (2) Cu(II) and Cd(II) metal complexes characterized by high thermal stability, good stability, excellent performance flame retardants, and wide application.

## Introduction

The structural features of four-membered N_2_P_2_ ring compounds in which the coordination number of P varies from three to five have attracted considerable attention^1,2^. Enhancing the thermal stability and flame retardancy of materials has been made possible by synthesizing several reactive or additive flame retardants by side-group substitution^3–5^. Moreover, flame retardants containing phosphorus and nitrogen have attracted much attention due to their high effectiveness and lack of toxicity. Furthermore, nitrogen and phosphorus can be combined to form more effective flame-retardant polymers to increase flame retardancy^6^. A few flame retardants based on nitrogen and phosphorus have been added to epoxy resin, and the compounds released after combustion are less hazardous^7^. Research on the potential applications of a recently discovered cross-linking polyphosphazene with active amine groups to reduce the fire hazard of epoxy resin was prompted by its successful synthesis^8^. Successful synthesis and characterization of hexasubstituted cyclotriphosphazene compounds with novel flame-retardant characteristics was achieved. Thermogravimetric analysis (TGA) and the limiting oxygen index were employed to investigate the flame-retardant property. Epoxy resin was used to create the molding matrix for this experiment^9^.

Phosphozenes and their derivatives have significant nitrogen and phosphorus contents, possibly contributing to their potential as flame retardants. Phosphazenes are superior building blocks for flame-retardant polymers^10–14^. They have been successfully used in epoxy and other resins^15,16^. Because of the strong reactivity between P-Cl bonds, hexachlorocyclotriphosphazene, or HCCP, is a flexible core molecule that readily undergoes nucleophilic substitution to generate cyclotriphosphazene derivatives.^17–19^. It is well known that cyclotriphosphazene, which consists of alternating nitrogen and phosphorus atoms, has high thermal stability^20,21^. Furthermore, the six-membered P-N ring of the substituted cyclotriphosphazene contributes to its chemical stability^22,23^. Interestingly, extensive studies using this molecule have led to its intended application in areas, such as flame retardants^24,25^. The remarkable properties of derivatives of cyclotriphosphazene have been used in the polymer industry to enhance the thermal and flame-retardant properties of polymers such as epoxy resin^26,27^. A cyclotriphosphazene-based epoxy synthesized by Seraji et al. was combined with diglycidyl ether of bisphenol F (DGEBF), an epoxy phenolic novolac resin^28^. A novel organic–inorganic hybrid polyphosphazene-modified manganese hypophosphite shuttle (PZS-MnHP) shuttle prepared to enhance the flame retardancy and anti-drip behavior of polyethylene terephthalate (PET). A comprehensive analysis of the flame-retardation procedure was also conducted^29^. A novel flame-retardant ingredient, hexachlorocylodiphosphazane, was physically added to epoxy varnish to provide both biocidal and flame-retardant properties. To evaluate any drawbacks associated with the additions, an analysis of the mechanical and physical resistances was also carried out^30–32^. Several arylhydrazone ligands and their Cu and Ni metal complexes have been discussed as potential flame retardant and antibacterial additives for polyurethane surface coatings^33–35^. A new additive based on a new sulphonamide ligand and its copper metal complex was added to a specific polyurethane varnish to make the coated film antibacterial and flame retardant^36^. In order to manufacture new flame-retardant paint, Cu(II) and Cd(II) metal complexes were prepared, and their efficacy was evaluated. The lowest possible oxygen percentage and ignitability were needed for combustion to continue^37^. Certain elements, such as nitrogen and boron, can combine with organophosphorus flame retardants to intensify their flame-retardant properties. Researchers have recently found that the synergistic effects of the chemical reaction between phosphorus and sulphur elements in polymer materials can rapidly increase the flame-retardant effects of polymer materials when organophosphorus flame retardants are prepared with sulfur elements in polymer polymerization^38–41^. The main objective of this study is the creation of novel substituted cyclodiphospha(V)zane derivatives to increase the exterior wood paint formulation flame-retardant properties. It was anticipated that this would make the material more resistant to ignition. Its flammability is also reduced with a limiting oxygen index (LOI) value. Furthermore, the effect of the type of alkyl chain length used on the mechanical and flame-retardant qualities is investigated.

## Experimental

### Materials and methods

High purity and analytical reagent grade (AR) chemicals were used in this study. Sulfadiazine, ortho nitro aniline, PCl_5_, copper chloride dihydrate (CuC1_2_·2H_2_O), cadmium chloride dihydrate (CdC1_2_·2H_2_O), diethyl ether, ethanol, and dimethyl sulfoxide (DMSO) were acquired from BDH Chemicals, UK. Merck products were used as indicators, including “perchloric acid, ammonia solution, ammonium chloride, nitric acid, ethylene-diaminetetraacetic acid disodium salt” (EDTA), Murexide, Eriochrome black-T (E.B.T), salicylic acid, and p-chloro-aniline.

### Instrumentation

After the complete degradation of the complexes, the metal contents were titrated against standard EDTA, and the metal concentration was calculated gravimetrically^42^. The phosphorus level was measured using phosphoammonium molybdate gravimetrically^43^. These micro-analytical determinations (C, H, N, Cl, and S) were performed at the Micro Analytical Center of Cairo University, Egypt. Using the KBr method, the IR spectra were recorded on a Perkin–Elmer 437 IR spectrophotometer (400–4000 cm^–1^). BurkerFTIR-400 MHZ spectrophotometer was used to measure the ^1^H and ^13^C NMR spectra, with TMS as the internal standard. A Sherwood Scientific Magnetic Susceptibility Balance” was used to record the solid-state magnetic susceptibilities of the complexes. Under a nitrogen environment, a “Shimadzu TGA-50H” with a flow rate of 20 mL min^−1^ was used for the thermogravimetric analysis. The UV–vis spectra were recorded using a “Perkin-Elmer Lambda 3B UV–vis spectrophotometer”, “Micro Analytical Centre, Cairo University.” Mass spectra were acquired using a direct “insertion probe” (DIP) and a “Shimadzu-Ge-Ms-QP 100-EX mass spectrometer” (Japan) within the temperature range of 50–1000 °C. Using MOE 2015 software, molecular docking studies were performed to examine the contacts and binding mechanism between the most active complexes and the proteins 5UHF and 4FM9^44^. The obtained results were compared with glycerol (GOL) and *N*-(2-methylphenyl)-Nalpha-(selenophene-2-carbonyl)-d-phenylalanin-amide (88D), reference compounds, using molecular docking models. The protein data bank (http://www.rcsb.org.pdb) was used to obtain the crystal structures of human topoisomerase II alpha (PDB ID: 4FM9) and M. tuberculosis (PDB ID: 5UHF). The 3D structures of the ligands, the most potent complexes, 88D and GOL, which were stored as MDL mol files, were constructed using Chem Draws 18.0. The molecule with the lowest binding affinity value was given the highest score.

### Synthesis of hexachlorocyclodiphosph(V)azane derivative H_2_L ligand

**Scheme 1:**
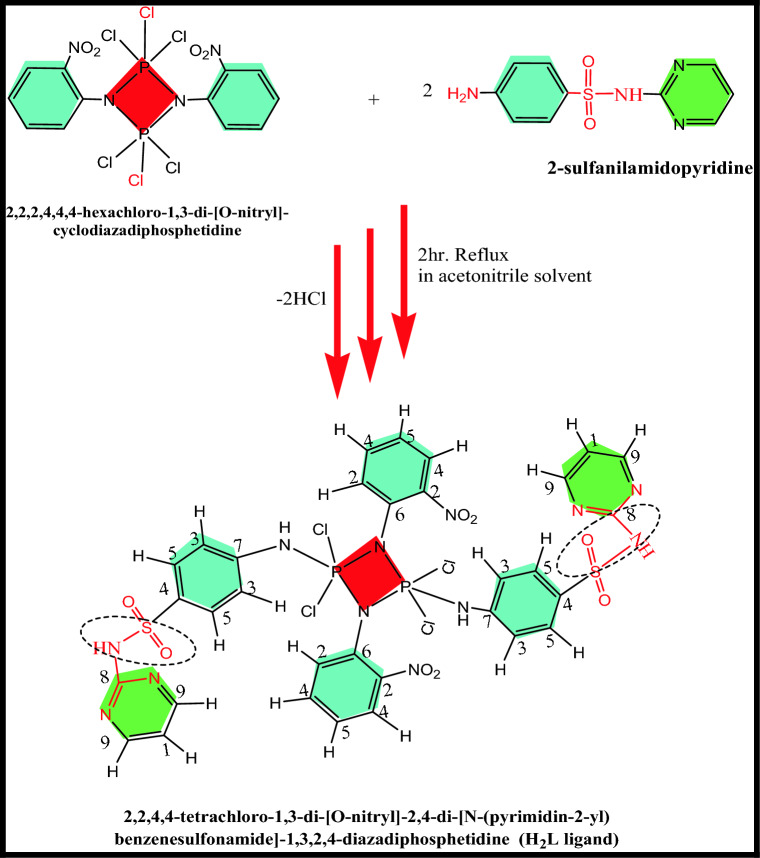
Synthesis of substituted cyclodiphospha(V)zane of the sulfa drug, H_2_L ligand.

The ligand H_2_L was prepared using the techniques of Zhumurova and Kirzanov^45^ and Chapman et al.^46^, as mentioned in the lecture work^47^. For 30 min, a well-stirred cold solution of ligand H_2_L (4.7 g, 0.01 mol) in 100 mL acetonitrile in a quick-fit flask was supplemented with a small amount of [*N*′-2-pyridinylsulfaniliamide] (5 g, 0.02 mol) in 100 mL acetonitrile after finishing the addition. The reaction mixture was refluxed for about two hours. The reaction was completed, and the (HCl gas stopped evolving). The resulting solid product was separated by filtration and sanitized by washing several times with acetonitrile and diethyl ether, then dried under vacuum over anhydrous CaCl_2_ to give the corresponding substituted hexachlorocyclodiphosph(V)azane H_2_L ligand (Scheme 1) m.p = 205 °C; Brown yellow, 41% yield (4 g).

### Metal complexes synthesis

After the successful preparation of the ligand, interest was focused on how it behaved with specific metal ions, including Cu(II) and Cd(II). The hot solution of H_2_L ligand (5 mmol) in 100 mL absolute ethanol was diluted dropwise with a hot solution of the aforementioned metal salts (10 mmol) in 50 mL absolute ethanol. The reaction mixture was refluxed for 2 h. The product underwent separation, then it was washed several times with ethanol and diethyl and then dried in vacuo.

## Results and discussion

### Characterization of the ligand

**Figure 1:**
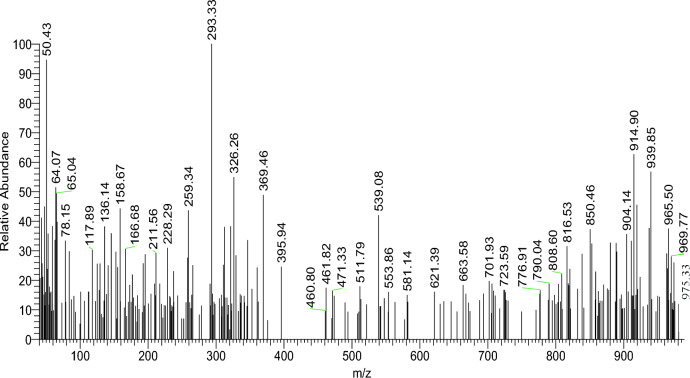
Mass fragmentation of H_2_L-free ligand.

The recently synthesized H_2_L ligand was subjected to elemental analyses, mass spectra, UV–vis, IR, and ^1^H, ^13^C NMR spectrum studies. The elemental analysis results (C, H, N, Cl, S, and P) accord well with the necessary values given by the suggested formulas. Scheme 1 and Table 1. The freshly synthesized ligand, H_2_L, exhibited a sharp melting point, indicating its purity. The molecular formula C_32_H_26_Cl_4_N_12_O_8_P_2_S_2_ is congruent with the mass spectrum, which revealed a base peak at m/z = 293.33 (100%), which is compatible with the molecular formula (C_6_H_11_CL_13_N_3_P_2_) and a molecular ion peak at m/z = 975.33 (12.82%), which is in good agreement with the proposed formula (Scheme 1). Figure 1. The UV–vis spectra of H_2_L-free ligand in 10^–3^ M with dimethylformamide (DMF) acting as a solvent. Table 2 displays the distinctive absorption band at 273 nm for the dimeric structure of the phosphorus four-membered ring^51^. The ligand spectrum displayed other absorption bands characteristic at 308 nm for π–π* transit transitions^52^.

**Table 1:** Elemental analyses and physical data of H_2_L ligand and its Cu(II) and Cd(II) metal complexes.

Compd. no. empirical formula (M.Wt)	M.P. (^o^c)	Color (yield %)	(%) found (Calcd.)							*Λ*m*
			C	H	N	Cl	S	P	M	
H_2_L C_32_H_26_Cl_4_N_12_O_8_P_2_S_2_ (975.33)	205	Brownyellow 41	39.44 39.00	2.69 2.70	17.2517.20	14.55 14.10	6.58 6.56	6.36 6 0.25	–	6.9
[(CuCl_2_)_2_(H_2_L)(H_2_O)_4_] C_32_H_34_Cl_8_Cu_2_N_12_O_12_P_2_S_2_ (1315.46)	< 300	Dark green 29	29.22 29.10	2.61 2.40	12.78 12.70	21.56 21.52	4.87 4.80	4.71 4.56	9.66 9.55	2.02
[(CdCl_2_)_2_(H_2_L)(H_2_O)_4_] C_32_H_34_Cd_2_Cl_8_N_12_O_12_P_2_S_2_ (1413.19)	< 300	Dark yellow 80	27.20 27.90	2.43 2.30	11.89 12.10	20.07 19.40	4.54 4.65	4.38 4.40	15,9 15.8	1.74

*ohm^−1^cm^2^mol^−1^.

**Table 2:** Electronic spectral data of H_2_L ligand and its Cu(II) and Cd(II) metal complexes.

Compound	Absorption bands (nm)					µeff (B.M.)	Geometry
	Phosphazo ring	π–π*	n–π*	*L-MCT*	*d-d* transition		
H_2_L	273	308	–	–	–	–	–
[(CuCl_2_)_2_(H_2_L)(H_2_O)_4_]	269	306	314	334, 396	666 (15,015), 706(14,164)	1.5	Octahedral
[(CdCl_2_)_2_(H_2_L)(H_2_O)_4_]	270	300	346	d^10^			Octahedral

Table 3, Fig. 3 present infrared data for the H_2_L ligand revealed distinctive vibration bands at 1155 cm^−1^ and 660 cm^−1^, which correspond to the υ (P-N) and υ (P-Cl) groups, respectively^21,53^. Bands located at 3359 cm^−1^ and 1620 cm^−1^ are ascribed to υ(NH) and υ(C=N), respectively^54^. The distinct proton signals were visible in the ^1^H-NMR spectra of the isolated ligand H_2_L. Table 4 and Fig. 3, recorded in DMSO-*d6* at room temperature. At 4.133, a wide signal was observed, indicating the (NH) proton of the enol/keto form of the H_2_L ligand, which can be exchanged with D_2_O^51^. On the other hand, a (–CH)hetro cyclic proton signal at δ = 8.48 ppm was proposed^55^. Furthermore, additional doublet-doublet signals for the H_2_L ligand were detected within the 6.59–7.99 ppm range attributable to aromatic protons^56^. The ^13^C NMR spectra of the H_2_L ligand revealed a peak at 40 ppm, which is attributable to DMSO-*d*6. The pyrimidine ringcarbon atom (C1) is assigned to the recorded signal at 115.84 and 116.07 ppm. Signals observed in the range (119.72–136.20) ppm are characteristic of different aromatic carbon positions, indicating distinct aromatic carbon locations^57^. The benzene carbon atoms attached to the nitrogen atoms of phospha(V)azo ring were assigned to the recorded signals at 146.62 and 149.06 ppm^58^. On the other hand, the remaining ^13^C signals at 157.57 and 158.76 ppm were due to (C=N) carbon atoms of the pyrimidine ring^58^, as seen in Table 5, Fig. 6.

**Table 3:** IR spectral bands (cm^−1^) for H_2_L-free ligand and its Cu(II) and Cd(II) complexes.

Comp no.	υ (NH)	υ(SO_2_) (asym.)	υ(SO_2_) (sym.)	υ (C=N)	υ (P–N)	υ (P–Cl)	υ(H_2_O) (Coord.)	υ(M–O)	υ(M–N)
H_2_L	3359sh	1343sh	1085sh	1620sh	1155sh	660sh	–	–	–
[(CuCl_2_)_2_(H_2_L)(H_2_O)_4_]	3354br	1322m	1092br	1585sh	1155sh	682m	841m	548m	410m
[(CdCl_2_)_2_(H_2_L)(H_2_O)_4_]	3355m	1322w	1092br	1585sh	1154m	684m	843w	569m	546m

sh, sharp; m, medium; br, broad; w, weak.

**Table 4:** ^1^H NMR data of H_2_L-free ligand and Cd(II) metal complex.

Chemical shift (δ in ppm)				
Comp.no	Aromatic protons	(–CH heterocyclic) protons	(–NH Exocyclic/–OH sulfonamide) protons	Coordinated water protons
H_2_L^1^	Pyrimidine Ring 6.585–7.017 Benzene Ring 7.382–7.990 Nitrobenzene ring 7.682–7.652	8.48(d,3H)	4.133 (s,2H)	–
[(CdCl_2_)_2_(H_2_L)(H_2_O)_4_]	Pyrimidine Ring 6.548–6.980 Benzene Ring 7.595–7.627 Nitrobenzene ring 7.608–7.637	8.50(d,3H)	3.93–4.60 (s, 2H)	3.173

**Table 5:** ^13^C NMR data of H_2_L-free ligand chemical shift (δ, ppm).

Types of carbon atoms	H_2_L
	Obs.
C1 (pyrimidine ring carbon)	115.84, 116.07
C2 (o-benzene ring carbon)	119.72
C3 (p-benzene ring carbon-SO2)	125.86
C4 (m-benzene ring carbon)	129.33, 130.14
C5 (p-benzene ring carbon)	136.20
C6, C7 (benzene ring carbon atoms attached to nitrogen atoms of phospha(V)azo ring)	146.62, 149.06
C8, C9 (pyrimidine ring (C=N) carbon atoms)	157.57, 158.76

### Structure and composition of metal complexes

In an attempt to clarify their molecular structures, new metal complexes of the H_2_L ligand of Cu(II) and Cd(II) were identified through elemental analyses (C, H, N, Cl, S, P, and M), molar conductance, electronic spectra Uv–vis, IR, ^1^H NMR, magnetic studies, thermal analyses TGA, and X-ray.

**Figure 2:**
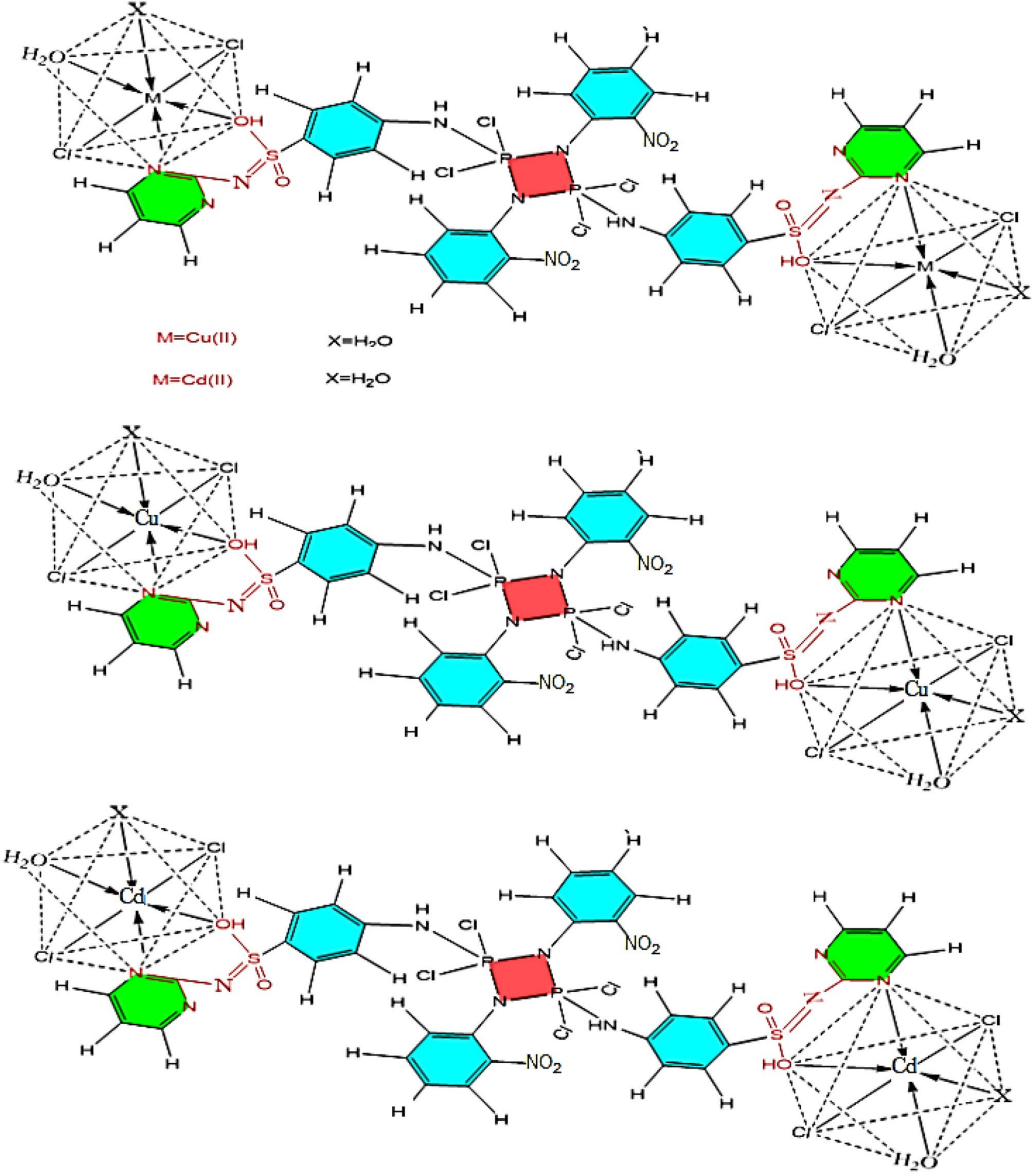
Suggested structures of Cu(II) and Cd(II) metal complexes.

The elemental analyses (C, H, N, Cl, S, and P) of the isolated Cu(II) and Cd(II) metal complexes are in good agreement with those required by the proposed formulae of the complexes Fig. 2.

Table 1 coincides fairly well with Fig. 2. The complexes are soluble in "DMSO" and "DMF" solvents but insoluble in water. The molar conductance (ΛM) studies of the complexes, which were performed at a concentration of 10^−3^ M using DMF as a solvent, show that the Cu(II) and Cd(II) complexes are not electrolytic^51^. The conductivity values were 2.02–1.74 Ω^−1^ cm^2^ mol^−1^, supporting their non-electrolytic behavior and neutrality of the coordination sphere^59^, as shown in Table 1. This finding confirms that the anions were inside the coordination sphere of the metal complex.

### Electronic spectrum and magnetic properties

Table 2 displays the electronic spectra of the metal complexes in (10^−3^ M) DMF at room temperature in the 200–800 nm wavelength range. Complexes show a characteristic absorption band similar to the phosphazo four-member rings assigned of H_2_L ligand, which is shifted to lower wavelengths at 269 nm and 270 nm for [(CuCl_2_)_2_(H_2_L)(H_2_O)_4_] and [(CdCl_2_)_2_(H_2_L)(H_2_O)_4_] complexes respectively^60,61^. For metal complexes, the absorption bands linked to the π–π* transitions are moved to shorter wavelengths at 306 nm and 300 nm. Additionally, the spectra of Cu(II) and Cd(II) complexes show a band at 314 nm and at 346 nm, respectively, which is linked to the n–π* transition^62^. Furthermore, the Cu(II) complex spectra show a band at 334 nm and at 396 nm linked to the (L–MCT) ligand to metal charge transfer^21,63^. Two bands at 666 nm (15,015 cm^−1^) and 706 nm (14,164 cm^−1^) exhibited a *d-d [*^*2*^*E*_*g*_* → *^*2*^*T*_*2g*_*(x*^*2*^*–y*^*2*^*)* and (^*6*^*A*_*1g*_* → *^*5*^*T*_*1g*_) transition for the Cu(II) complex, which suggested an octahedral environment^64,65^. Also, an octahedral environment for the copper complex was confirmed by the reported magnetic moment value of 1.5 B.M^59^. Moreover, an octahedral geometry is most likely postulated for the Cd(II) complex because d-d transitions are not expected for such a filled d^10^ system, and the observed bands for the Cd(II) complex are due to intra-ligand transitions and revealed a diamagnetic property^66,67^.

### IR spectra and bonding mode

**Figure 3:**
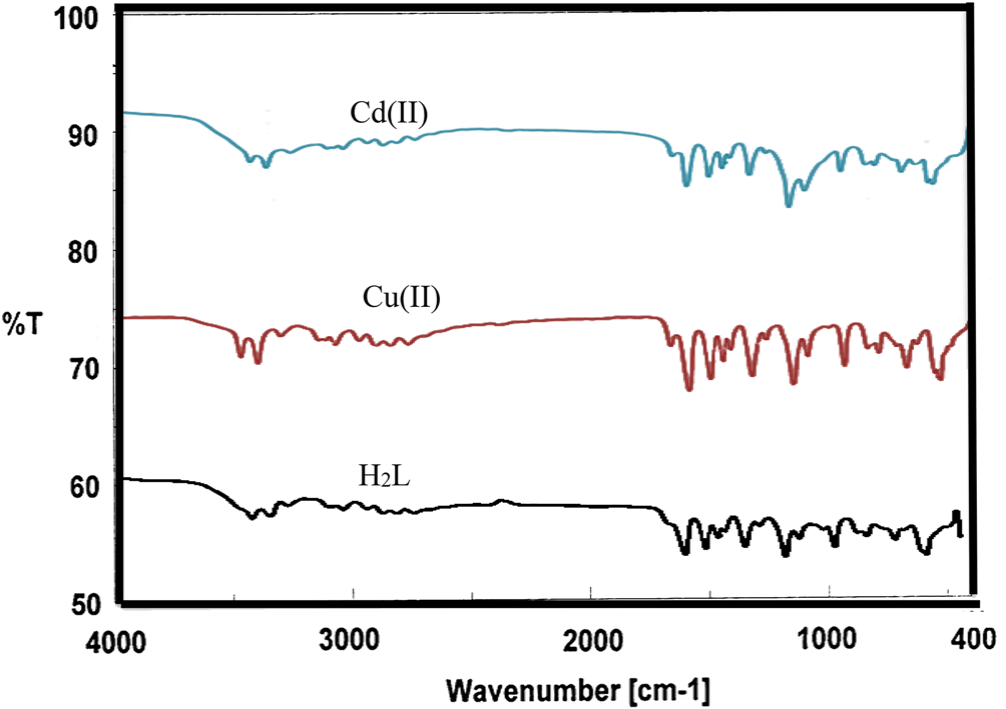
IR spectra of H_2_L-free ligand and its Cu(II) and Cd(II) complexes.

The infrared spectra of the freshly synthesized H_2_L ligand and those of its Cu(II) and Cd(II) complexes, shown in Table 3 and Fig. 3, were obtained in the range of 4000–400 cm^−1^ and assisted in identifying absorption regions caused by the corresponding vibrations. It demonstrates that the spectra of complexes displayed absorption bands at 682 cm^−1^, 684 cm^−1^, 1155 cm^−1^, and 1154 cm^−1^ are attributed to υ(P–Cl) and υ(P–N) for Cu(II) and Cd(II) complexes respectively^53,64^.

The IR spectra of the complexes were contrasted with those of the free ligand to identify the most probable coordination sites involved in chelation. Chelation was intended to have an impact on the location and intensity of these peaks. The sulfonamide group's υ(NH) was discovered at 3359 cm^−1^. According to a careful examination of the free ligand and its Cu(II) and Cd(II) metal complexes, they were hidden behind the wide bands, and new absorption broad bands of the enolic ʋ(OH) appeared at 3354 cm^−1^ and 3355 cm^−1^ in the spectrum of the isolated Cu(II) and Cd(II) complexes for the free ligand^51^. For the H_2_L ligand, the sulfone group in the double bond stretching region asym (O=S=O) and sym (O=S=O) were present at 1343 cm^−1^ and 1085 cm^−1^. Following interaction with Cu(II) and Cd(II), the transition metal ions in these sulfone group bands were relocated to lower frequencies at 1322 cm^−1^ and 1322 cm^−1^, respectively, than the ligand H_2_L. The red shift of the SO_2_ band to higher frequencies at 1092 cm^−1^ can be attributed to the change of the sulfonamide group (–SO2NH) to the enol form (–SO(OH)=N) as a result of complex formation to build a more stable six-membered ring^64^. The spectra also indicated a sharp band due to the stretched vibration rings of diazine (C=N), which occurred at 1620 cm^−1^ for the H_2_L and then was shifted to lower frequencies changed to 1585 cm^−1^ in the Cu(II) and Cd(II) Complexes spectra^68^. This finding suggests that the coordination via* the* pyrimidine-N atom of the heterocycle ring –N is involved in the production of complexes^64^. Coordinating water infrared bands (H_2_O) appeared at 841–843 cm^−1^ for metal complexes, indicating water molecules bonding to metal ions^59^. Thermal gravimetric analyses were used to demonstrate the coordinated nature of the water molecules. Additionally, there are two additional bands at 548–569 cm^−1^ and 410–546 cm^−1^ attributed to the ν(M–O) and ν(M–N) stretching vibrations, respectively, which are explained by the IR spectra of Cu(II)and Cd(II) metal complexes^64^. Form the above, it is evident that the H_2_L ligand coordinated with metal ions through two sites, the enolic OH of the sulfonamide group and pyrimidine-N.

### ^1^H NMR spectra

**Figure 4:**
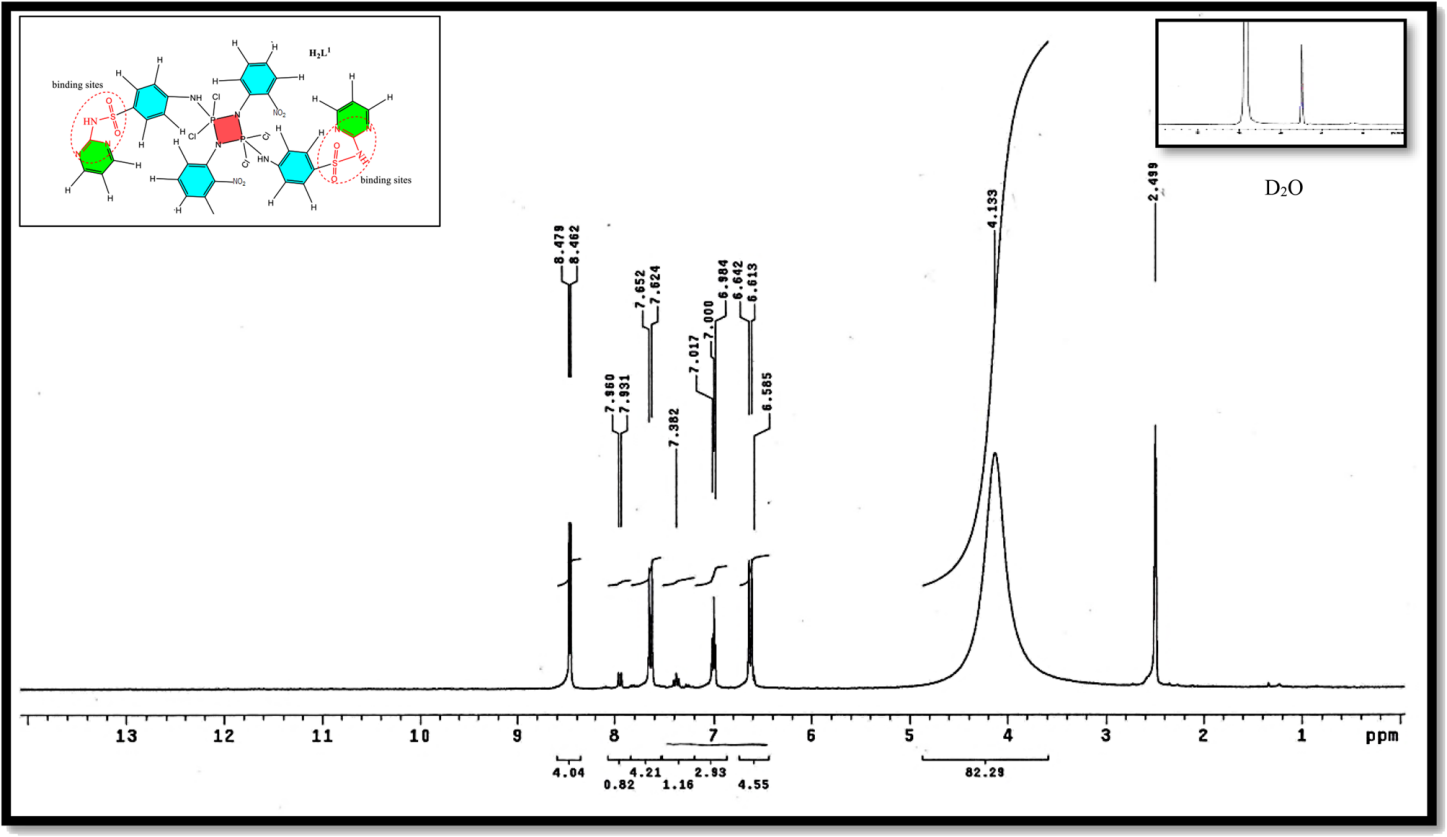
^1^H NMR spectrum of H_2_L-free ligand.

**Figure 5:**
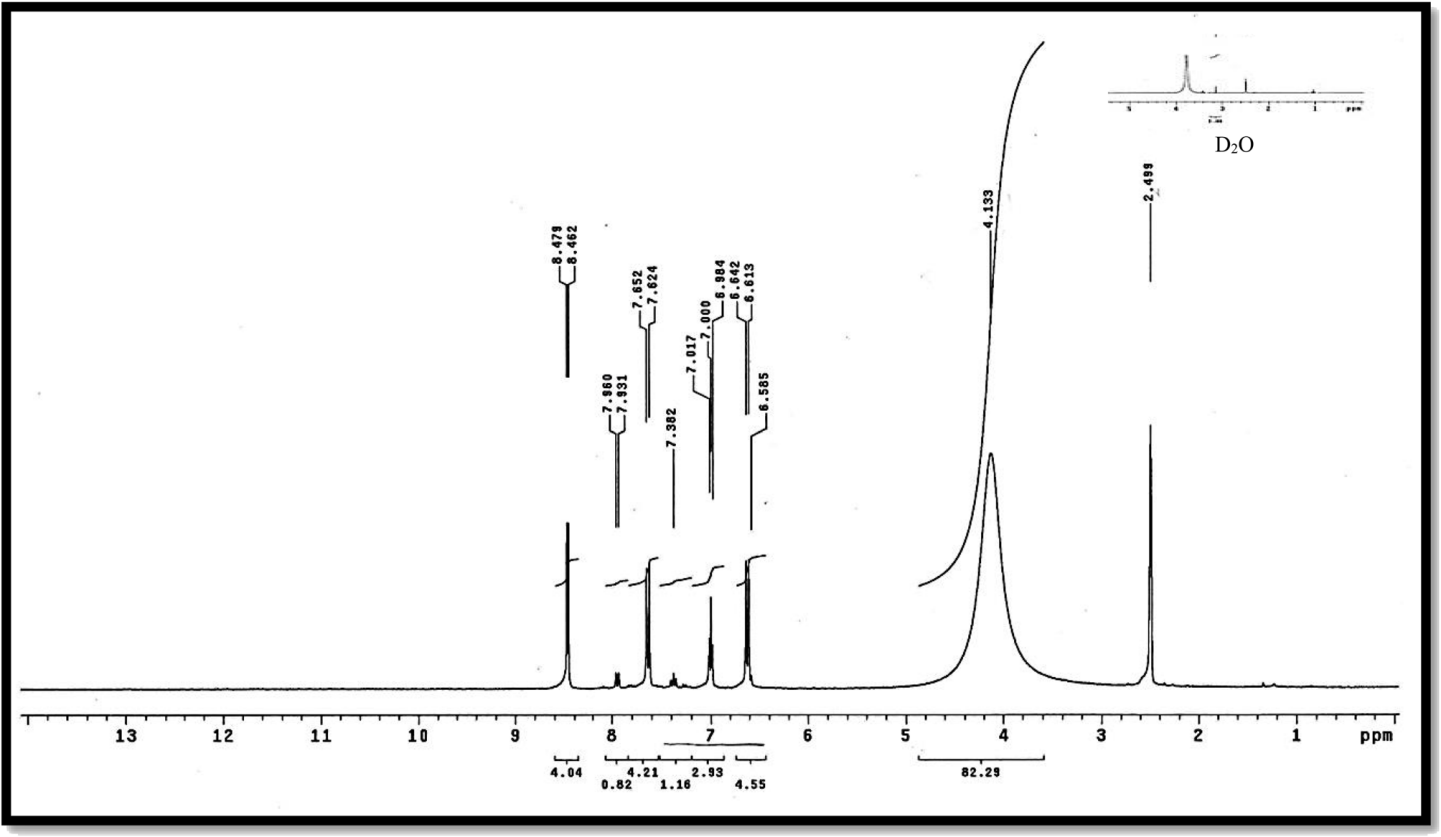
^1^H NMR diagram of Cd(II) of H_2_L-free ligand.

**Figure 6:**
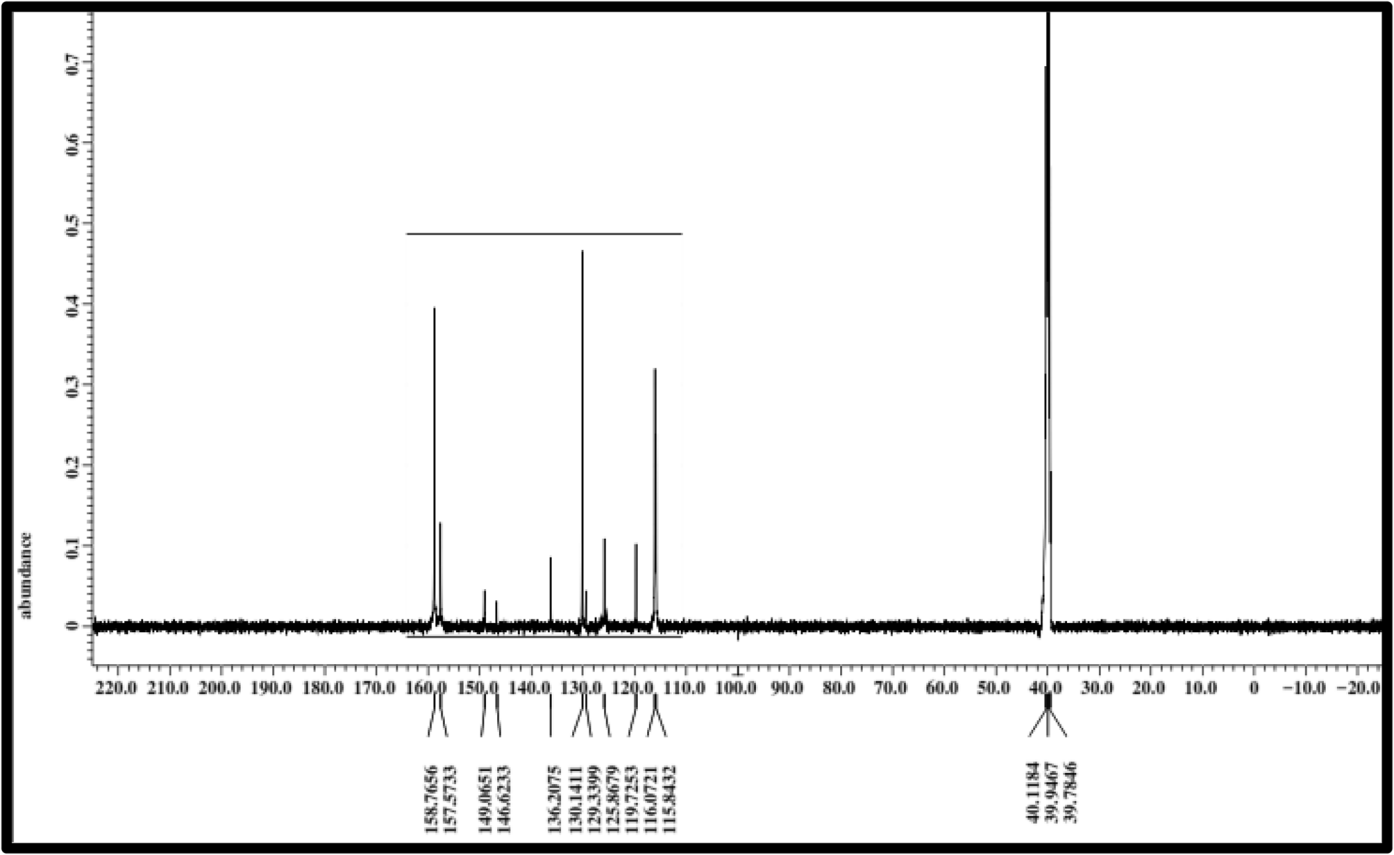
^13^C NMR data of H_2_L-free ligand.

Upon complexation using DMSO*d*_6_ as solvent, the ^1^H NMR spectra of Cd(II) complexes in Table 4 and compare the distinctive proton signals of the Cd(II) complex to those of the parent H_2_L ligand in Figs. 4, 5, 6. This shows new broad enolic(–SO(OH) = N–) signal at δ = 3.93–4.60 ppm was observed, which confirms the involvement of the sulfonamide group in the complexation^69^. Additionally, for diamagnetic complexes, the aromatic protons multi-signals first occurred at δ = 6.55–7.64 ppm^62^. For Cd(II) complexes, a new signal was discovered in the ring at δ = 3.17 ppm under complexation, corresponding to coordinated water molecules^21,24^.

### Thermogravimetric analyses (TGA)

**Figure 7:**
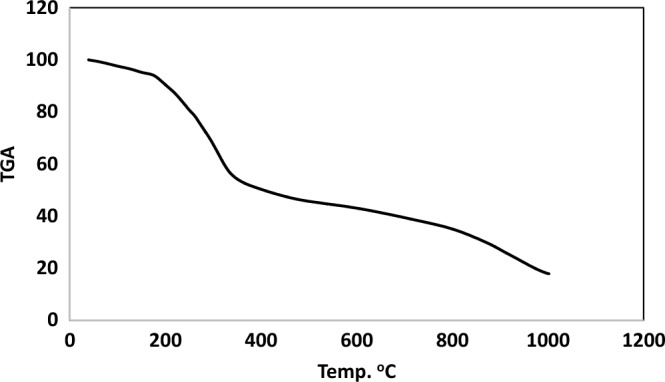
TGA cruve of Cu(II) complex.

Water molecules are categorized as either coordinated within the inner coordination sphere or crystalline outside of it in Cu(II) and Cd(II) metal complexes, as determined by thermogravimetric analyses (TGA). These analyses also describe the thermal breakdown process of these chelates^70^. Table 6 provides a proof of the data, and the weight loss of each chelate was computed within the relevant temperature ranges. Within the temperature range of 38–1000 °C, TGA of the [(CuCl_2_)_2_(H_2_L)(H_2_O)_4_] complex of H_2_L-free ligand exhibits five stages of decomposition, as indicated by the TGA curves. The initial phase occurs between 38 and 137 °C (Fig. 7), resulting in the decomposition of 4H_2_O (Coordinated), with a measured mass loss of 5.80% (calcd. 5.47%). The second phase involves an approximate mass loss of 4.21% (calcd. 4.13%) with a temperature range of 137–224 °C loss of 2HCN. The third decomposition stage, occurring between 224 and 517 °C, is the loss of the organic part C_10_H_8_Cl_4_N_4_P_2_S_2_O_4_ with an estimated mass loss of 39.19% (calcd. 39.92%). The fourth stage, conducted at temperatures of 517–848 °C, involves the loss of organic compound C_10_H_12_Cl_3_N_3_O_2_, resulting in a mass loss of 24.92% (calcd. 23.71%). Finally, the fifth stage at the temperature 848–1000 °C resulted in the decomposition of the organic part C_10_H_7_N_4_ and HCl with a found mass loss of 16.61% (calcd. 16.6%), leaving a metallic residue of 2CuO. The total weight loss is 90.65% (calcd. 88.77%).

**Table 6:** Thermogravimetric of Cd(II) and Cu(II) complexes.

Complex	Temp. range (^°^C)	n*	Loss in weight Estim./(calcd.)%		Loss in weight Estim./(calcd.)%	Metallic residue
			Mass loss Total mass loss			
[(CuCl_2_)_2_(H_2_L)(H_2_O)_4_]	38–137	5	5.80 (5.47)	90.6(88.77)	-Loss of 4H_2_O	2CuO
	137–224		4.21(4.13)		-Loss of 2H CN	
	224–517		39.19(38.92)		-Loss of C_10_H_8_CL_4_N_4_O_4_ P_2_S_2_	
	517–848		24.92(23.71)		-Loss of C_10_H_12_ Cl_3_ N_3_ O_2_	
	848–1000		16.61(16.6)		-Loss of C_10_H_7_N_4_ + HCL	
[(CdCl_2_)_2_(H_2_L)(H_2_O)_4_]	39–156	3	5.22 (5.09)	81.97(82.91)	-Loss of 4H_2_O	2CdO
	156–462		47.72(48.83)		-Loss of C_20_H_13_Cl_4_O_2_ N_4_P_2_S_2_	
	462–1000		29.09(29.06)		-Loss of C_12_H_13_Cl_4_ N_8_O_4_	

n*, number of decomposition step.

**Figure 8:**
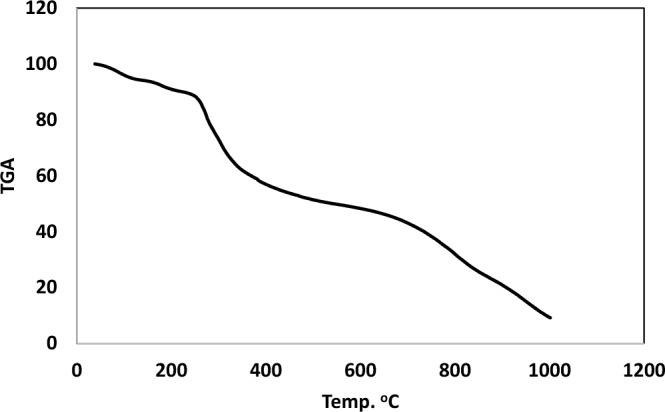
TGA curve of Cd(II) complex.

Moreover, the TGA analysis curves of the [(CdCl_2_)_2_(H_2_L)(H_2_O)_4_] metal complex of H_2_L-free ligand revealed three decomposition stages occurring between 39 and 1000 °C Fig. 8. The first stage, beginning at 39–156 °C, involves the removal of four coordinated 4H_2_O molecules, causing mass loss of 5.22% (calcd 5.09%). The second stage occurred at 156–462 °C; there was a mass loss of 47.71% (calc. 47.83%) due to the removal of HCl and the organic parts C_20_H_13_Cl_4_N_4_O_2_P_2_S_2_. During the third stage, at a temperature of 462–1000 °C, a mass loss of 29.09% (calcd. 29.06%), resembling the elimination of the organic parts C_12_H_13_Cl_4_N_8_O_4_. The stable 2CdO metallic residue is the ultimate product. Meanwhile, the net weight loss was 81.97% (calcd. 82.91%).

According to these study results, we proposed a structure for the newly prepared ligand and its metal complex as presented in Scheme 1 and Fig. 2.

### X-ray diffractometer

**Figure 9:**
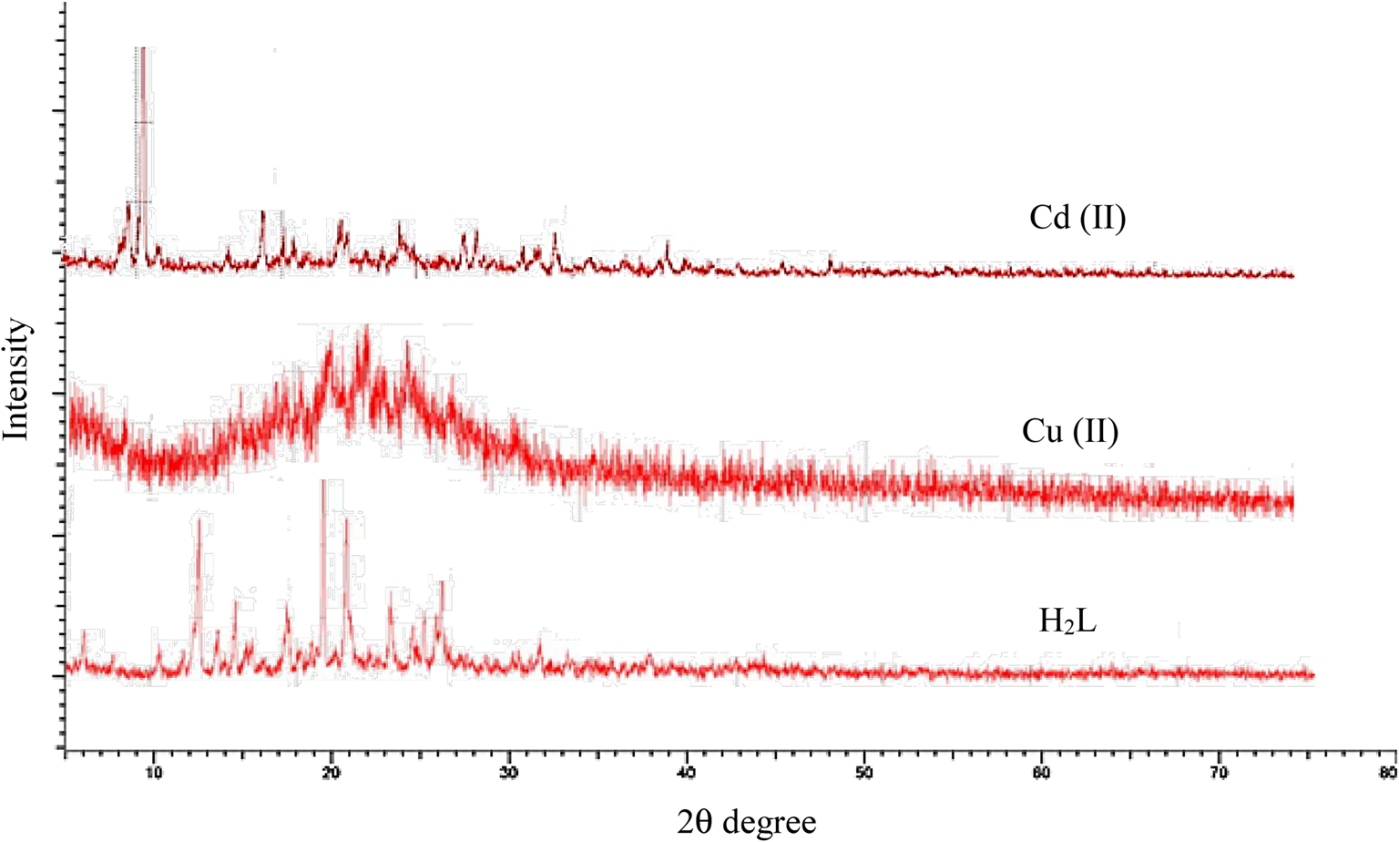
X-ray powder diffractogram of the H_2_L-free ligand, Cu (II), and Cd(II) metal complexes.

A powder X-ray diffractometer was used to determine the crystalline structure of the synthesized H_2_L-ligand and its [(CuCl_2_)_2_(H_2_L)(H_2_O)_4_]and[(CdCl_2_)_2_(H_2_L)(H_2_O)_4_] complexes. Figure 9 displays the powder XRD patterns of the ligand and metal complexes. Their sharp peaks indicated their crystalline structure^36^. Metal ions cause variances in X-ray patterns between the metal complexes, which are crystalline, and their original ligand. The average crystal size of the samples was calculated using the Debye–Scherrer equation. The formula D = K λ/βCosθ where β is the full-width half maximum (FWHM) of the characteristic peak, θ is the Bragg diffraction angle for the hkl plane, and λ is the wavelength of the used X-ray source, which is 1.54 Å, K is a constant equal to 0.94^36,71^. The average crystalline size of the free H_2_L-ligand Cu(II) and Cd(II) metal complexes was 9.7–5.6 and 8.7 nm, respectively.

### Docking investigation

**Figure 10:**
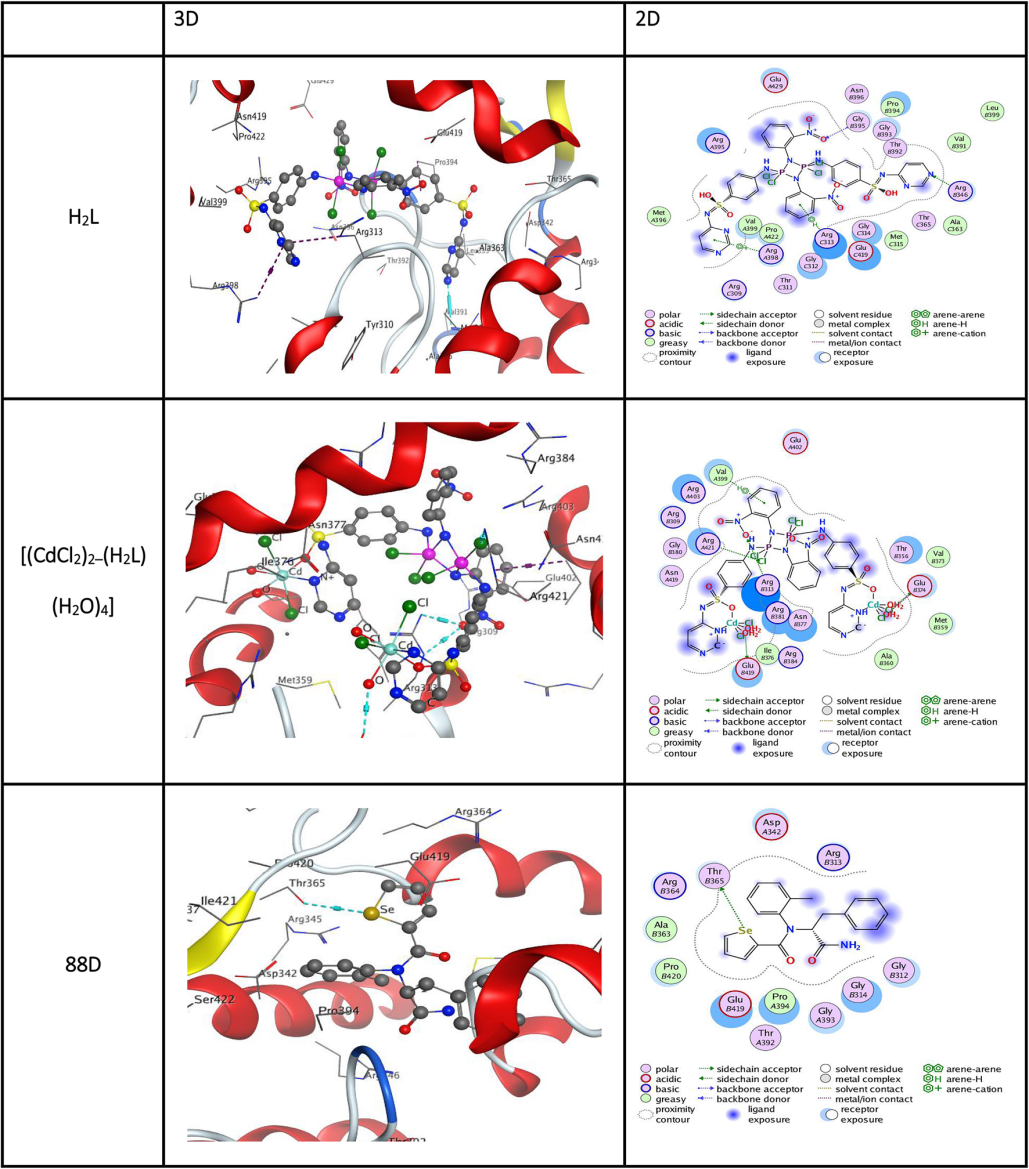
2D & 3D interaction of H_2_L and 88D in the active site of M. tuberculosis (PDB ID: 5UHF). Hydrogen bonds are displayed in cyan and H-pi-bonds in dark magenta.

**Figure 11:**
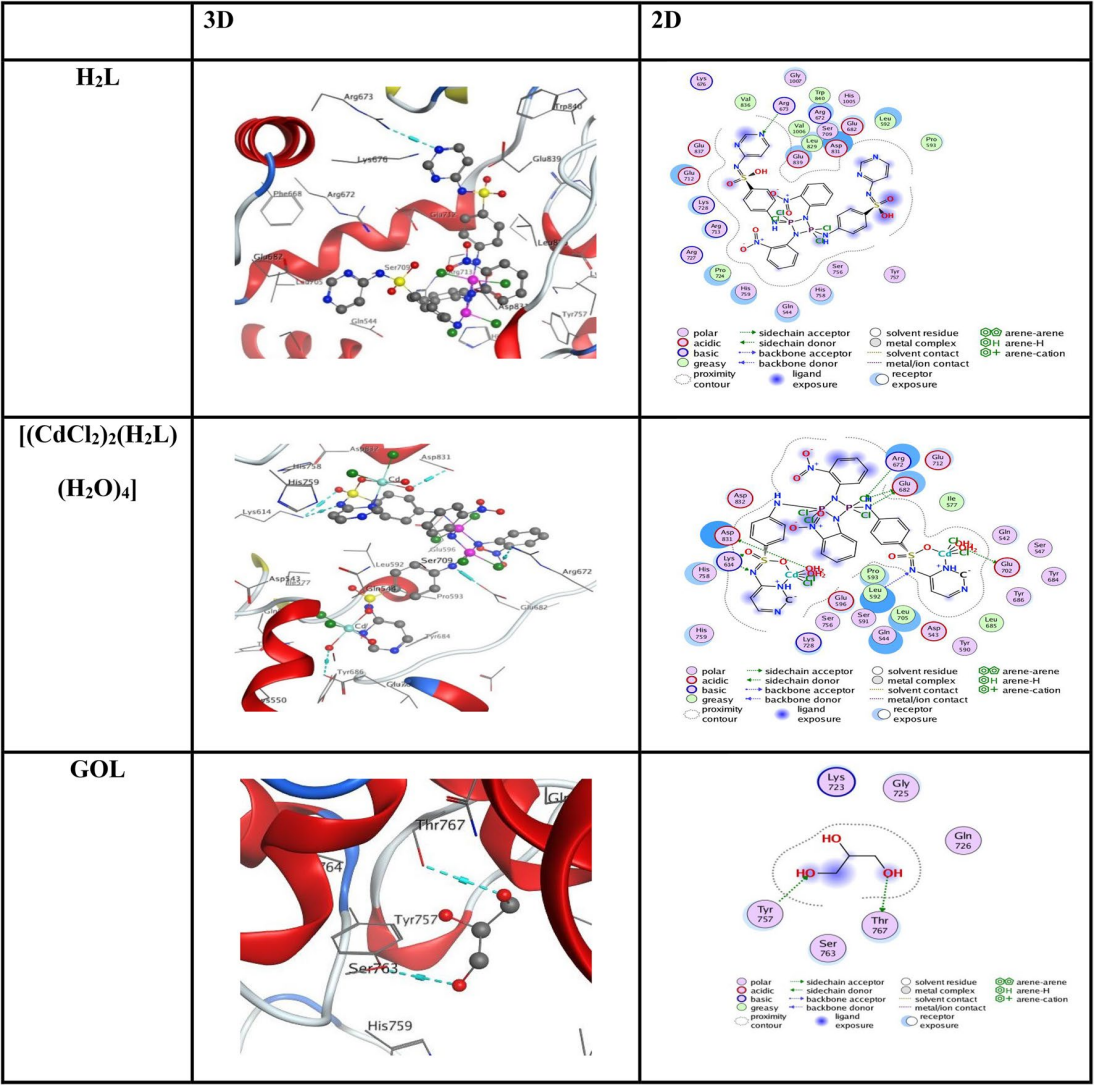
2D & 3D interactions H_2_L,[(CdCl_2_)_2_(H_2_L)(H_2_O)_4_], and GOL in the active site of human topoisomerase II alpha (PDB ID: 4FM9). Hydrogen bonds are displayed in cyan.

Docking was performed on the *M. tuberculosis* receptor (PDB ID: 5UHF) using the proteins 5UHF with MOE 2015 software to assess the docking activity of the most potent produced compounds H_2_L, [(CdCl_2_)_2_(H_2_L) (H_2_O)_4_]), and the control (88D). The docked molecules had a higher binding affinity (− 6.10 kcal mol^−1^) than the control 88D, which is greater. The docking energy scores for the metal-complexes of and docked ligands were determined to be − 8.48, − 7.76, and − 9.25 kcal mol^−1^ for the (a) H_2_L and [(CdCl_2_)_2_(H_2_L) (H_2_O)_4_] complexes, respectively (Table 7). The negative binding energy increases in proportion to the intensity of the interaction. The interaction occurred in the order of H_2_L > [(CdCl_2_)_2_(H_2_L) (H_2_O)_4_]. These results are consistent with the examination of antibacterial efficacy. Figure 10 illustrates the bonding interactions between the docked molecules and 88, including hydrogen bonds and polar and hydrophobic interactions with particular amino acid residues in the 5UHF protein. The MOE 2015 software analyzed the most potent compounds, H_2_L and [(CdCl_2_)_2_(H_2_L)(H_2_O)_4_], together with the control GOL, concerning the human topoisomerase II alpha receptor docking (PDB ID: 4FM9). The docked molecules exhibit a higher binding affinity of − 3.76 kcal mol^−1^ when compared to the control GOL. The docking energy score for the metal complexes and docked ligands in the (a) H_2_L and [(CdCl_2_)_2_(H_2_L) (H_2_O)_4_] complexes was determined to be − 12.45 and − 9.13 kcal mol^−1^, respectively, as shown in Tables 7 and 8. The stronger the engagement, the higher the negative energy score. The interaction proceeded according to the pattern H_2_L > [(CdCl_2_)_2_(H_2_L) (H2O)_4_]. These conclusions align with the anticancer properties observed in the investigation. Figure 11 displays the total bonding interactions among the amino acid residues in the 4FM9 protein, the docked molecules, and GOL, encompassing hydrogen bonds and polar and hydrophobic interactions.

**Table 7:** Docking results of H_2_L, [(CdCl_2_)_2_(H_2_L)(H_2_O)_4_], and 88D inside *M. tuberculosis* (PDB ID: 5UHF) active spots.

	Ligand	Receptor	Interaction	Distance (in A^o^ from main residue)	E (Kcal mol^−1^)	S (Kcal mol^−1^)
H_2_L	N 43	ARG 346	H-acceptor	3.25	− 4.7	− 9.25
	O 85	GLY 395	H-acceptor	3.12	− 2.2	
	6-ring	ARG 398	pi-cation	4.21	− 0.6	
	6-ring	ARG 313	pi-H	3.78	− 0.9	
[(CdCl_2_)_2_(H_2_L)(H_2_O)_4_]	Cl 38	GLU 374	H-donor	2.83	− 1.8	− 7.76
	O 57	GLU 419	H-donor	2.72	− 1.4	
	Cl 85	ARG 421	H-acceptor	3.26	− 0.9	
	O 104	ARG 313	H-acceptor	3.33	− 0.8	
	O 104	ARG 313	H-acceptor	2.90	− 1.8	
	O 36	GLU 374	ionic	3.82	− 0.9	
	O 57	GLU 419	ionic	2.72	− 6.6	
	6-ring	VAL 399	pi-H	4.20	− 0.8	
88D	Se 25	THR 365	H-donor	3.72	− 1.0	-6.10

**Table 8:** Docking results of H_2_L, [(CdCl_2_)_2_(H_2_L)(H_2_O)_4_], and GOL inside human topoisomerase II alpha (PDB ID: 4FM9) active spots.

	Ligand	Receptor	Interaction	Distance (in A^o^ from main residue)	E (Kcal mol^−1^)	S (Kcal mol^−1^)
H_2_L	N 28	ARG 673	H-acceptor	3.48	− 1.9	− 12.45
[(CdCl_2_)_2_(H_2_L)(H_2_O)_4_]	O 39	ASP 831	H-donor	3.25	− 2.5	
	O 57	GLU 702	H-donor	3.44	− 0.7	
	Cl 85	GLU 682	H-donor	3.17	− 0.3	
	N 89	GLU 682	H-donor	2.84	− 6.2	
	N 23	LYS 614	H-acceptor	3.38	− 0.8	
	O 34	LYS 614	H-acceptor	3.32	− 1.7	
	N 41	LEU 592	H-acceptor	3.08	− 2.4	
	Cl 85	ARG 672	H-acceptor	3.08	− 0.9	
	O 39	ASP 831	ionic	3.25	− 3.0	
	O 57	GLU 702	ionic	3.44	− 2.1	− 8.73
	O 13	THR 767	H-donor	3.05	− 1.1	-3.76
GOL	O 1	TYR 757	H-acceptor	3.24	− 0.6

The activity of ligands and their metal complexes as antibacterial and anticancer agents was demonstrated by docking them against PDB IDs 5UHF and 4FM9. The substances exhibit significant protein binding and numerous interactions.

### Application of the prepared cyclodiphosph(V)azane Ligand H_2_L and its metal complexes as flame-retardant additives

#### Coating composition and their flame retardant evaluation

Cyclodiphosph(V)azane ligand H_2_L and its Cu(II) and Cd(II) metal complexes were added to the formulation of wood paint at a ratio of 1.0% to prepare the coating compositions. Table 9 lists the varnish composition in tabular form. The samples with different molar ratios were then applied to the wood panels using a brush. Every attempt was made to keep the film thickness constant at 60 ± 5 µm to assess its mechanical and physical characteristics.

**Table 9:** Composition of the exterior wood paint formulation.

Component	Wt.%
Long soya alkyd 70%	44
Disperse 601	0.35
Benton 34	0.3
Methanol	0.55
Terpentine	5.0
TiO_2_	17
C0-Octoate	0.4
Ca-Octoate	0.4
Zr- Octoate	0.48
Anti skinning	0.5
Intermediate	31
Ultramarine blue	0.02
Flame retardant additives Co (II), Ni (II) metal complexes	1.0

Specific gravity: 0.90 ± 0.03, Theoretical spreading: 17.0–10.0 M2/LTR (Depends on wood and method of application), touch dry 4–6 h *, dry to recoat 12–16 h dry to overcoat 24 h.

#### Physical and mechanical testing of coating films

The preparation of the steel panels followed the ASTM D: 609 standards. Moreover, the dry film thickness was measured according to the ASTM D: 1005 standard. While film hardness was measured according to the ASTM D523 standard in measuring the gloss level. The ASTM D: 3363 standard was followed in measuring the film hardness. The adhesion and flexibility were tested using ASTM D: 3359 and ASTM D: 522, respectively, while the resistance to mechanical damage, or “impact resistance,” was determined using ASTM Method D: 2794-93.

##### The specimens' mechanical and physical characteristics, both painted and unpainted

The mechanical and physical characteristics of the coating were influenced by adding cyclodiphosph(V)azane and its Cu(II) and Cd(II)) complexes and these effects were evaluated following certain standards. This was conducted to determine if adding the chemicals had any adverse consequences. The examined attributes were adhesion, flexibility, cross hatch, scratch toughness, and gloss. Relevant outcomes were provided. The results in Table 10 indicate that adding metal complexes substantially enhanced gloss, cross-hatch adhesion, hardness, impact resistance values, and chemical resistance^39–72^.

**Table 10:** Physical and mechanical characteristics of the coated exterior wood films based on the prepared flame-retardant additives.

Formulation	FR %	Gloss at 60^◦^C	Hardness (Kg)	Adhesion	Impact (J)
Blank Wood paint formulation	–	85	1.5	4B	1.3
Wood paint formulation & Ligand (H_2_L)	0.5	90	2.0	5B	1.7
	1.0	95	2.4	5B	2.0
Wood paint formulation & Cu(II) complex	0.5	90	2.2	5B	1.9
	1.0	96	2.4	5B	2.2
Wood paint formulation & Cd(II) complex	0.5	90	2.3	5B	1.9
	1.0	96	2.5	5B	2.2

Additionally, the prepared cyclodiphosph(V)azane derivative enhanced the mechanical properties of the dry-painted films, including the gloss levels, when used as the basis for the synthesized Cu(II) and Cd(II) complexes. Cross-hatch adhesion levels range from 4B to 5B, hardness by pencil (kg) (1.5–2.5), and impact (J) ranges from 1.3 to 2.2. Furthermore, the metal complexes identified can improve the accuracy of this test in comparison to the blank sample, which lacks manufactured additives like ligands and its Cu and Cd metal complexes. The favorable outcomes can be attributed to the complex structure that includes aromatic rings, P, N, Cu, and Cd, which improve the mechanical characteristics.

#### Thermal and ignition characteristics of the painted and unpainted tasters

##### Ignitability

Time to ignition and heat release:

The TTI and THR are crucial parameters to consider when assessing the flammability and flame retardancy of interior materials^73^. This test was conducted in compliance with ASTM E176^48,49^. Table 11 demonstrates the TTI of coated plywood samples with their Cu(II) and Cd(II) metal complexes, both in their original state (blank sample) and after being treated with a phosphazene ligand. The findings revealed that the coated wood sample, treated with a wood paint formulation blended with phosphazene ligand and its Cu(II) and Cd(II) metal complexes, had the longest TTI compared to other samples, including uncoated plywood sample and coated samples mixed only with cyclophoshazene ligand. The inclusion of the cyclophosphazene ligand and its Cu(II) and Cd(II) metal complexes was observed to improve the TTI and THR. These improvements of incorporated paint formulation with either cyclophosphazene ligand or its Cu(II) and Cd(II) metal complexes may be attributed to (1) firstly according to the incorporation with the cyclophosphazene ligand, this is due to the presence of synthesized phosphorus, various elements of nitrogen and chlorine atoms, (2) the presence of Cu(II) and Cd(II) metal complexes beside the synthesized phosphorus, various elements of nitrogen, which form an excellent intumescent carbon layer with high mechanical characteristics and thermal stability. Moreover, results indicated that the heat flux increased from 10 to 25 kW m^−2^, causing all specimens to ignite with different ignition periods ranging from 550 to 1200 s, respectively. This suggests that the uncoated wood sample burns more readily. Furthermore, when compared to a coated plywood sample containing incorporated paint mixed with a manufactured phosphazene ligand and its Cu(II) and Cd(II) metal complexes to the burnt specimen, the latter would have shattered and emitted significant smoke due to the emission of CO and CO_2_.

**Table 11:** Features of flame retardant of the uncoated plywood sample and coated by formulation of paint based on prepared phosphazene ligand H_2_L and its Cu(II), Cd(II) metal complexes as flame retardant additives.

Formulation	FR (%)	Ignitability		Limiting oxygen index (LOI) (%)
		Heat flux (kW m^−2^)	Ignition time (s)	
Uncoated sample	0.0	10	550	21
Coated sample without additives	0.0	10	760	26
Coated sample based on Cyclophosphazen ligandH_2_L	1.0	15	980	90
Coated sample based on Cu(II) metal complexes of Cyclophosphazene ligand	1.0	20	1200	130
Coated sample based on Cd(II) metal complexes of Cyclophosphazene ligand	1.0	20	1200	130

##### Limiting oxygen index

Using rheometric scientific equipment, the minimum oxygen concentration in a mixture of nitrogen and oxygen needed to maintain specimen ignition was determined following the standard^50^.

The flame-retardant properties of the wood paint were assessed by using phosphazene derivatives and their Cu(II) and Cd(II) metal complexes through the LOI test. A higher oxygen concentration requirement indicates a more flame-resistant sample. The test findings demonstrated that involving phosphazene ligand and its Cu(II) and Cd(II) metal complexes in the wood paint formulation, as indicated in Table 11, enhanced the flame-retardant properties of the coating in comparison to a blank sample. Table 11 indicates that samples coated with wood paint mixed with cyclophosphazene ligand or its metal complexes had flame retardant times ranging from 90 to 130 s, respectively. In contrast, the blank and plywood-coated samples without these additives had LOI values of 21% and 26%, respectively. Due to the presence of about 21% oxygen in the atmosphere, a material with a lower LOI than 21% would readily combust in the air. A material with an LOI value between 21 and 28% is considered “slow-burning.” Additionally, a substance is considered self-extinguishing when the flame or ignition source is eliminated. The incorporation of the phosphazene ligand and its Cu(II) and Cd(II) metal complexes increased the LOI value of the blended wood paint system. The alkyd thermoset polymer network in the paint formulation comprises the phosphazene ligand along with Cu(II) and Cd(II) metal complexes to improve the paint flame-retardant properties. Due to the presence of nitrogen, chlorine, and phosphorus along with Cu(II) and Cd(II) in phosphazene compounds, they exhibit increased fire behavior compared to blank samples because of the same response presented previously (the presence of Cu(II) and Cd(II) metal complexes beside the synthesized phosphorus, various elements of nitrogen, which form an excellent intumescent carbon layer with high mechanical), thereby providing improved flame-retardant features. The burnt samples are all depicted in Image  3, which included the blank sample, the coated sample without generated ligand, and the coated sample with paint combined with the synthesized compounds.

**Image 1:**
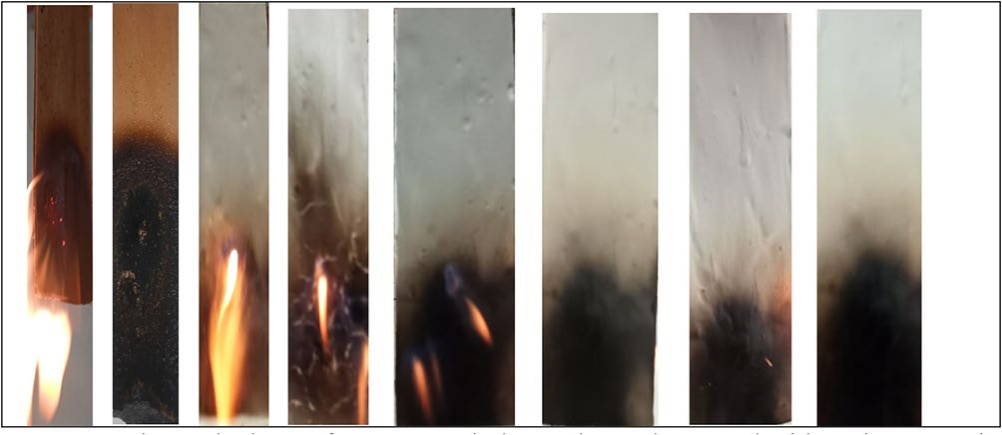
Shows the burn of an uncoated plywood sample, coated without incorporation with prepared compounds, and coated based on prepared H_2_L-ligand its Cu(II) and Cd(II) metal complexes.

#### Flame-retardant mechanism^74–76^

1. Mechanism using halogen (chlorin) in the synthesis of phosphazene derivatives.

The flame-retardant functions by absorbing heat as the material heats up, releasing denser than air hydrogen halide gas on the material surface to remove oxygen and generating active free radical HO during the combustion in the material's gas phase. The flame-retardant effect is achieved by reacting with hydrogen halide, forming stable and inert free radicals. Halogenated flame retardants disrupt the chain reaction by preventing hydroxide and hydrogen free radicals from interacting with oxygen and carbon monoxide. Combustion is impeded by the trapping of radicals, which interrupts the chemical reaction of the exothermic oxidative flame. Here are the mechanisms of the reactions in the vapor phase.

- H^**.**^ + HX = H_2_ + X
- HO^**.**^+ HX = H_2_O + X
- RH + X^**.**^= R^**.**^ + HX

2. Mechanism by using phosphorus in the prepared phosphazene derivatives.

Organophosphorus flame retardants typically operate through the following mechanism: (1) When the system of flame-retardant crashes, volatile radicals (PO, PO2, etc.) are released into the gas phase. These radicals can trap active radicals (H and HO) and stop the combustion chain reaction. The breakdown of organophosphorus flame retardants will simultaneously produce a non-combustible gas diluted into the surrounding air, reducing the oxygen level. (2) The breakdown and condensation of organophosphorus flame retardants in the condensed phase facilitates the matrix dehydration and carbonization to form a carbon layer. Moreover, it can develop a glass-like molten coating on the combustion surface upon thermal decomposition, which further inhibits heat and gas transmission, resulting in a flame-retardant effect. Combining certain elements, such as boron, nitrogen, etc., with organophosphorus flame retardants might enhance their flame-retardant attributes. Recent studies suggest that the flame-retardant properties of polymer materials can be rapidly increased by creating organophosphorus flame retardants that include sulfur-based components. The synergistic effect results from the interaction between the phosphorus and sulfur components in the polymer substance. Thus, organophosphorus compounds, such as phosphazene, are mostly employed in intumescent paints and coatings. In addition, foams that undergo expansion are created by intumescent systems. Because of this property, they safeguard flammable materials like wood and plastic and materials such as steel that degrade under high temperatures.

3. Mechanism using nitrogen-based flame retardants through the synthesized phosphazene derivatives.

Nitrogen-based flame retardants are particularly interesting due to their low toxicity, less smoke emission in flames, and excellent flame-retardant efficiency. Nitrogen flame-retardants produce non-flammable gases during combustion that can impede gas phase combustion and generate a flame-retardant effect. Reduce oxygen and gaseous fuel levels by generating NH_3_ or N_2_ while heating, allowing them to diffuse gradually into the combustion zone. Nevertheless, flame retardants containing nitrogen have a restricted amount of nitrogen. The material lacks both compatibility and flame-retardant efficacy.

### Future prospects of our synthesized compounds

The cyclotriphosphazene-based chemicals would enable further investigation into the flame retardant of steel, wood, and textiles. This is because flame retardant coating applications and cyclotriphosphazene-based flame retardants have excellent chelating performance.

The potential applications of our synthesized compounds beyond wood paint can be promising as anticorrosive materials for steel protection when incorporated with primer coating formulation in the future. The effectiveness of cyclotriphosphazene and its metal complexes encourages future corrosion inhibitors, along with anticorrosive coating applications, to have excellent with epoxy resins, and with different polymers which would be important in the study of corrosion inhibition. Also, the prepared compounds can be used as insecticide agents and antimicrobial additives materials for protective coating,^77–80^**.**

## Conclusions

The synthesized ligand H_2_L and its metal complexes were separated and characterized using chemical analysis, mass spectra, conductivity measurements, electronic spectrum data UV–vis, FT-IR, ^1^H,^13^C-NMR, TGA, XRD, and docking investigations. The prepared cyclophosphazene derivatives and their Cu(II) and Cd(II) metal complexes were incorporated into the paint formulations for coating plywood samples. Further research was carried out to assess the flame-retardant properties of the coated samples using ignition time and LOI tests. The synthesized chemicals included in the experiment enhanced the maximum LOI value and improved the flame-retardant quality of the coated formulation. When combined, nitrogen and other elements can boost the flame-retardant attributes of organophosphorus flame retardants. Organophosphorus flame retardants can be synthesized by leveraging the synergistic effects of the chemical reaction between phosphorus and Sulfur elements in the polymer materials. This process rapidly enhances the flame-retardant properties of polymer materials.

## Data Availability

The datasets used and analyzed during the current study are available from the corresponding author upon reasonable request.
